# Effects of Acute Coordinative vs. Endurance Exercise on Cortisol Concentration in Healthy Women and Men

**DOI:** 10.1186/s40798-025-00884-z

**Published:** 2025-06-10

**Authors:** Henning Budde, Mirko Wegner, Christiane Ahrens, Bruna Velasques, Pedro Ribeiro, Sergio Machado, Thomas Gronwald, Sandra Amatriain-Fernández, Marcelle Schaffarczyk, Anett Mueller-Alcazar

**Affiliations:** 1 Institute for Systems Medicine (ISM), MSH Medical School Hamburg, University of Applied Sciences and Medical University Hamburg, Hamburg, Germany; 2 Department of Health Science, Institute of Human Movement Science, University of Hamburg, Hamburg, Germany; 3 Bioscience Department, School of Physical Education of the Federal University of Rio de Janeiro, Rio de Janeiro, Brazil; 4 Institute of Applied Neuroscience, Rio de Janeiro, Brazil; 5 Brain Mapping and Sensory Motor Integration, Institute of Psychiatry, Rio de Janeiro, Brazil; 6 Panic and Respiration, Institute of Psychiatry, Federal University of Rio de Janeiro, Rio de Janeiro, Brazil; 7 Laboratory of Physical Activity Neuroscience, Neurodiversity Institute, Queimados-RJ, Brazil; 8 Institute of Interdisciplinary Exercise Science and Sports Medicine, MSH Medical School Hamburg, Hamburg, Germany; 9 G-Lab, Faculty of Applied Sport Sciences and Personality, BSP Business and Law School, Berlin, Germany; 10 Institute of Sport Science, Friedrich Schiller University Jena, Jena, Germany; 11 Institute for Cognitive and Affective Neuroscience, MSH Medical School Hamburg, University of Applied Sciences and Medical University Hamburg, Hamburg, Germany

**Keywords:** Exercise, Coordinative, Endurance, Cortisol, Acute, Intensity

## Abstract

**Background:**

Physical exercise can cause neuroendocrine activation, which increases salivary cortisol concentrations. Until now there have been no studies comparing the acute effects of endurance and coordinative exercise on cortisol.

**Objective:**

To examine the effects of an acute bout of endurance vs. coordinative exercise with the same intensity and duration with an intraindividual comparison.

**Methods:**

61 female and male students between 18 and 30 years completed an acute coordinative exercise (Co) and one week later an endurance exercise (En) of the same exercise intensity, which was self-set on the first day, with a heart rate of between 64–76% of maximum over a period of 15 min. To measure changes in the hypothalamic–pituitary–adrenal axis activity, saliva samples were collected before (t0 and after exercise (t1: 5 min, t2: 30 min).

**Results:**

Baseline values of cortisol (t0) did not differ significantly (t(55) = .233, *p* = .816). Analysis of variance revealed main effects for type of exercise (*F* (1) = 5.587, *p* = .022*,* η^2^ = .092) and measurement point (*F* (2) = 22.472, *p* < *.001,* η^2^ = .290) as well as an interaction effect of the two factors (*F* (2, 110) = 4.322, *p* = .016*,* η^2^ = .073). Post hoc tests indicated that in the Co group the cortisol t2 values differed significantly from t0 to t1. In the En group, however, t1 differed significantly from t0 to t2. Moreover, cortisol levels differed significantly between Co and En at t2 (t(55) = 3.661, *p* = .004).

**Conclusion:**

For the first time we showed that Co produced a higher cortisol release than En of the same exercise intensity and duration. Interventions such as Co require higher cognitive engagement resulting in higher cortisol than En.

**Trial Registration:**

DRKS, Deutsches Register Klinischer Studien. Registered 20 July 2020, https://drks.de/search/de/trial/DRKS00016590.

## Key Points

- Cortisol is an important catabolic hormone in relation to overtraining or burnout.
- Our results show for the first time that acute coordinative exercise produces a higher cortisol release than endurance exercise of the same exercise intensity and duration.

## Background

Acute physical exercise influences neuroendocrine responses and can lead to an increase in cortisol concentration if the intensity of the intervention exceeds a certain threshold [1–3]. Physical exercise (PE) refers to targeted, planned and structured forms of physical activity (PA) [4]. PE should be differentiated into acute PE (single bout) and chronic PE (repeated bouts of PE or physical training) [4]. Studies suggest that enhanced chronic and regularly conducted PA facilitates neuroplasticity of certain brain structures and is associated with an improvement in neurogenesis, synaptogenesis, and the release of neurotrophins and neuroendocrinological changes that are linked to benefits for cognitive and affective functions [5]. In the long term, this may be reflected in a reduction in psychosocial stress symptoms [6] and in short term led to an improvement in cognitive performance [7].

In the event of acute physical or psychological stress, our body’s stress response essentially occurs via two axes. The sympatho-adrenomedullary system puts the body into an alarm state when it is exposed to stress and ensures an increase in heart and respiratory rate by releasing adrenaline and noradrenaline [8]. The hypothalamic–pituitary–adrenal axis (HPA-axis] forms the second stress axis, which is primarily characterized by the release of the stress hormone cortisol [9]. Cortisol has multiple effects on metabolism [10], the immune system and inflammatory processes [9], and is the most important hormone of the HPA-axis alongside corticotropin-releasing hormone (CRH) and adrenocorticotropic hormone (ACTH). Acute physical exercise is acknowledged as an immediate stressor for the body and affects the secretion processes of several endocrine tissues and the subsequent release of hormones like cortisol [11].

The circadian rhythm of cortisol is stable and is only slowly influenced by external conditions. During acute physical stress, a peak secretion of cortisol can be observed approximately 20 to 30 min after the cessation of exercise [12]. If the organism is unable to terminate the stress response, e.g., in the case of chronic stress exposure, stress-adaptive mechanisms can lead to pathological changes. The role of the endocrine stress system can be seen in the pathophysiology of various mental illnesses such as depression [6], burnout [13], or post-traumatic stress disorder [14]. Also, physical exercise can induce changes in the functioning of the HPA-axis, provided that a certain intensity threshold of PA is exceeded [6, 15].

The intensity and duration of the PA are considered the primary factors for determining the neuroendocrine stress response [11]. Adult subjects showed a linear increase in salivary cortisol concentration over a period of 15 min at a maximum exercise intensity of 70–85% maximum heart rate (HRmax), whereas no significant changes were observed at lower exercise intensities [15]. In connection with psychosocial stress, the cortisol response appears to be even more pronounced [16]. It can therefore be assumed that, in addition to the intensity of the stressors, the type of stressor itself also has an influence on cortisol levels and that psychosocial stress can increase cortisol levels significantly more than purely physical stress of moderate intensity (65–75% HRmax) [17].

Salivary cortisol responses show great intra- and inter-individual variability and are determined by various factors [18]. Personality traits, age, sex, endogenous and exogenous sex steroid levels, genetic predispositions or critical life events and in particular negative childhood experiences are worth mentioning in this context [19]. Several studies have shown that chronic endurance training (which is defined as planned, structured, repetitive, and purposeful PA leading to a change in fitness) [4, 20], can effect cortisol concentration in the long term [21] and thus has both a preventive and therapeutic effect in reducing the experience of stress [22]. This shows that high levels of PA are related to a lower physiological stress reactivity. The immediate neuroendocrine stress responses induced by physical exercise are not yet fully understood. Results of studies comparing different types of chronic PE interventions showed that coordinative exercise led to a greater reduction in cortisol concentrations and stress levels compared to chronic endurance exercise [21]. However, the immediate stress responses produced by acute PE interventions are still not clear, especially regarding how the acute responses differ depending on the type of exercise performed. Different types and modes of acute PE have gained increased attention in research in recent years [23].

Based on current research, the question of whether there are direct effects of different acute interventions of the same exercise intensity (i.e., with the same cardiovascular load) on the cortisol concentration will therefore be investigated. A distinction is made between acute physical exercise of a coordinative nature and acute physical endurance exercise. As higher cognitive abilities will be required during the coordinative exercise (e.g. the ability to react and concentrate) [24], both physiological and psychological stressors are at work here. For this reason, it is assumed that stress load will be higher. Thus, the type of acute physical stress could be an essential factor in determining the neuroendocrine stress response. To our knowledge, there have been no studies on whether cortisol concentration differs between an acute coordinative exercise and an acute endurance exercise of the same exercise intensity and duration. The reaction to physical interventions is demonstrably individual and depends on many influencing factors [25]. For this reason, the intra- and inter-individual variability should be given special consideration here [23] and influencing factors such as sex and PA will be investigated exploratively.

## Methods

### Participants

The G*Power analysis (α = 5%, 1-β = 95%, d = 0.95) resulted in a required sample size of 60. The participants were informed on the characteristics of their participation, agreed to participate and signed the informed consent form. The study was approved by the ethics committee of the MSH Medical School Hamburg (MSH-2021/131) and was conducted in accordance with the latest revision of the Declaration of Helsinki (2013). Inclusion criteria for participation in the study were the absence of mental or physical impairment, drug or alcohol dependence, and acute psychotic illness. Based on these criteria, no participant had to be excluded. In addition, we asked the participants, who were recruited from the local universities, about their perceived social support and their satisfaction with their everyday social relationships and measured their self-perceived stress. Motor fitness and PA were also assessed. A total of 61 people between the ages of 18 and 29 took part in the study; 32 were female. The average age was 21.8 years (SD = 3.1, N = 61).

### Study Design

For the present study, a moderate exercise intensity just before the vigorous range was aimed for, below on Garber et al.'s classification of exercise intensity [26]. Therefore, the intensity of the different exercise sessions should be between 64 and 76% HRmax. Participants' HRmax was calculated according to Tanaka’s et al.’s formula: HRmax = 208—(0.7 × age) [27], providing a maximum anchor or prescribing exercise intensities for both acute interventions. Additionally, we assessed the rate of perceived exertion (RPE) using the Borg scale, a numerical scale between 6 and 20 to record the subjective feeling of exertion [28]. The HR was continuously recorded and monitored with a chest strap device and a HR monitor (Polar H10 and M430, Kempele, Finland) before and during each intervention.

After registering, the participants were instructed not to exercise for 24 h and not to eat for two hours before the start of the exercises. Each participant took part in two interventions, which took place between 2 p.m. and 4 p.m. seven days apart. In the first session, questionnaires were used to collect demographic data and information on PA as well as the current state of loneliness which took about 20 min. During this time, participants were able to calm down from possible distractions of the day. A demographics questionnaire included questions about physiological parameters (height, age and weight) and the subject’s personal circumstances. The Godin Leisure-Time Exercise Questionnaire (GLTQ) [29] was used to assess PA. The GLTQ determines the level of PA and has been shown to correlate with the level of physical fitness [30]. The Questionnaire to assess the Motor Function State—Fragebogen zur Erfassung des motorischen Funktionsstatus (FFBmot) [31] was used to assess motor fitness. The subscales of the FFBmot are the components strength, endurance, flexibility and coordination. In addition, before the second intervention (En), the subjects’ subjective experience of loneliness was assessed using the UCLA Loneliness Scale [32].

After, a coordination exercise (Co) lasting 15 min was initially performed using a coordination ladder which was elevated 10 cm off the floor, the subjects were asked to complete various step and jump sequences. The coordination ladder consisted of ten squares (45 × 45 cm), which were demarcated by poles and attached loosely to cones. During the coordination exercise, the participants had to concentrate on not knocking down the poles. In the first round, the participants ran loosely above the ladder and familiarized themselves with the distances and heights of the obstacles. The second round began with the jump sequences, which increased in complexity with each round and required an increasing level of cognitive performance. The highest level consisted of jumping diagonally through the course on one leg. After eight minutes, the level of difficulty was slowly reduced again. At the end of the coordinative intervention, the participants had to perform a balance exercise (standing scale) for 20 s on each leg.

After a washout phase of seven days, the second intervention was carried out at the same time at the same indoor spot with the same group sizes of a maximum of six in form of endurance exercise (En) with the same intensity for the cardiovascular system. The standardized individual target HR and the RPE of the Co determined the intensity of the En. Again, the subjects first completed a questionnaire in order to acclimatize for about 20 min. After attachment of the HR measuring devices, the subjects had to run for 15 min, while receiving tactile feedback about the HR level they should achieve. RPE was again assessed using the Borg scale [28].

The planned study had a quasi-experimental, “pre-post-intervention” design with repeated measurements [33]. The intraindividual variability of the cortisol concentration describes the different responses of the same subject to two different interventions at three different measurement times. To standardize the intensity of En, Co had to be performed first, and randomization of the subjects was therefore not possible. The reason the study design was not randomized is because of the difficulty of ensuring comparable exercise intensity levels between En and Co. It is very demanding to control Co intervention with a pre-defined HR and/or RPE.

The first saliva sample for the measurement of cortisol concentration was taken immediately before the intervention (t0), the second sample five minutes after completion (t1) and the third sample 30 min after the end of the exercise (t2). Saliva samples were collected using the SaliCap system (IBL Hamburg) and stored at minus °C until laboratory analysis. The saliva analysis method is considered a reliable non-invasive way of determining stress-related adrenal hormone levels [34]. The concentration of cortisol in the saliva correlates closely with the concentration of free cortisol in the blood, and it has been confirmed that the measurement in the saliva is a reliable tool for investigations of HPA activities [35]. The biochemical analysis was carried out at the University of Technology Dresden using immunoassay analysis. Intra- and inter-assay coefficients of variation were 1.8 and 2.9%, respectively.

### Data Analysis

Due to non-evaluable cortisol samples, two participants had to be excluded. Three others did not complete the exercise with the required intensity and were also excluded. The sample for the calculation comprised 56 participants (25 male and 31 female).

For two of the six measurement points a total of 5 samples were missing. Overall, this was 1.5% missing data which allowed for imputation techniques. Multiple imputation techniques were employed using SPSS to complete the data set using the mean of five different random models. As a result, complete cortisol values for all six measurement points and all 56 participants could be analyzed. For further analysis the cortisol values were log-transformed because they were not normally distributed.

The main analysis of the changes in cortisol release after both interventions (Co, En) was carried out using an ANOVA with repeated measures. Data were tested for sphericity and if present corrected using the Mauchly test. Effect sizes were calculated in the ANOVAs, for significant results also Eta-square (η^2^) values are reported.

In addition, the influences of the variables sex, motor fitness (surveyed by questionnaire FFBmot) and PA (questionnaire GLTQ) on the differences between the individual measurements was exploratively examined and considered as covariates in the context of the ANOVA. The influences of sex (*F* (1,50) = 0.959, *p* = *0.332,* η^2^ = 0.019), motor fitness (FFBmot, *F* (1,50) = 1.91, *p* = *0.172*, η^2^ = 0.037), and PA (*F* (1,50) = 0.029, *p* = *0.866*, η^2^ = 0.001) were not significant. Furthermore, the measured cortisol concentrations were not influenced by any of these variables. The subjective feeling of loneliness (UCLA) also showed no influence on the measurement results (F (1,50) = 0.022, *p* = *0.883*, η^2^ = 0.001). Therefore, the following analyses for cortisol were reported and analysed without any covariate. Thus, to test the differential effects of Co vs. En exercise we ran repeated-measures ANOVAs with post-hoc test with Holm correction for multiple testing.

## Results

Preliminary analyses suggest that none of the demographic variables (age, sex, BMI, PA, mood) affected the results significantly and were thus not included as covariates in the ANOVA (see Data Analysis). Furthermore, baseline values of cortisol (t0) did not differ significantly between Co and En (t (55) = 0.233, *p* = 0.816). During Co, participants’ mean HR was within the predefined HR target (142.2 ± 3.0 bpm, 73.8 ± 1.7% HRmax) with a RPE of 13.9 ± 0.6. The mean HR during En was comparable to Co (142.5 ± 2.8 bpm, 74.0 ± 1.6% HRmax, *t* (55) = 0.562, *p* = 0.576, *d* = 0.075) with a RPE of 13.8 ± 0.8.

Mean values and standard deviations for cortisol concentrations are shown in Table 1.

**Table 1:** Means (M) and standard deviations (SD) for corticol concentrations (nmol/l) at pre- (t0) and post-test I (t1) and II (t2) for the coordinative exercise (Co) and endurance exercise (En) groups [n = 56]

Measurement	Co		En	
	M	SD	M	SD
*Cortisol* (nmol/l)				
Pre (t0)	3.52	2.69	3.24	1.98
Post I (t1)	3.24	2.42	2.54	1.45
Post II (t2)	5.41	5.73	3.70	2.53

**Fig. 1:**
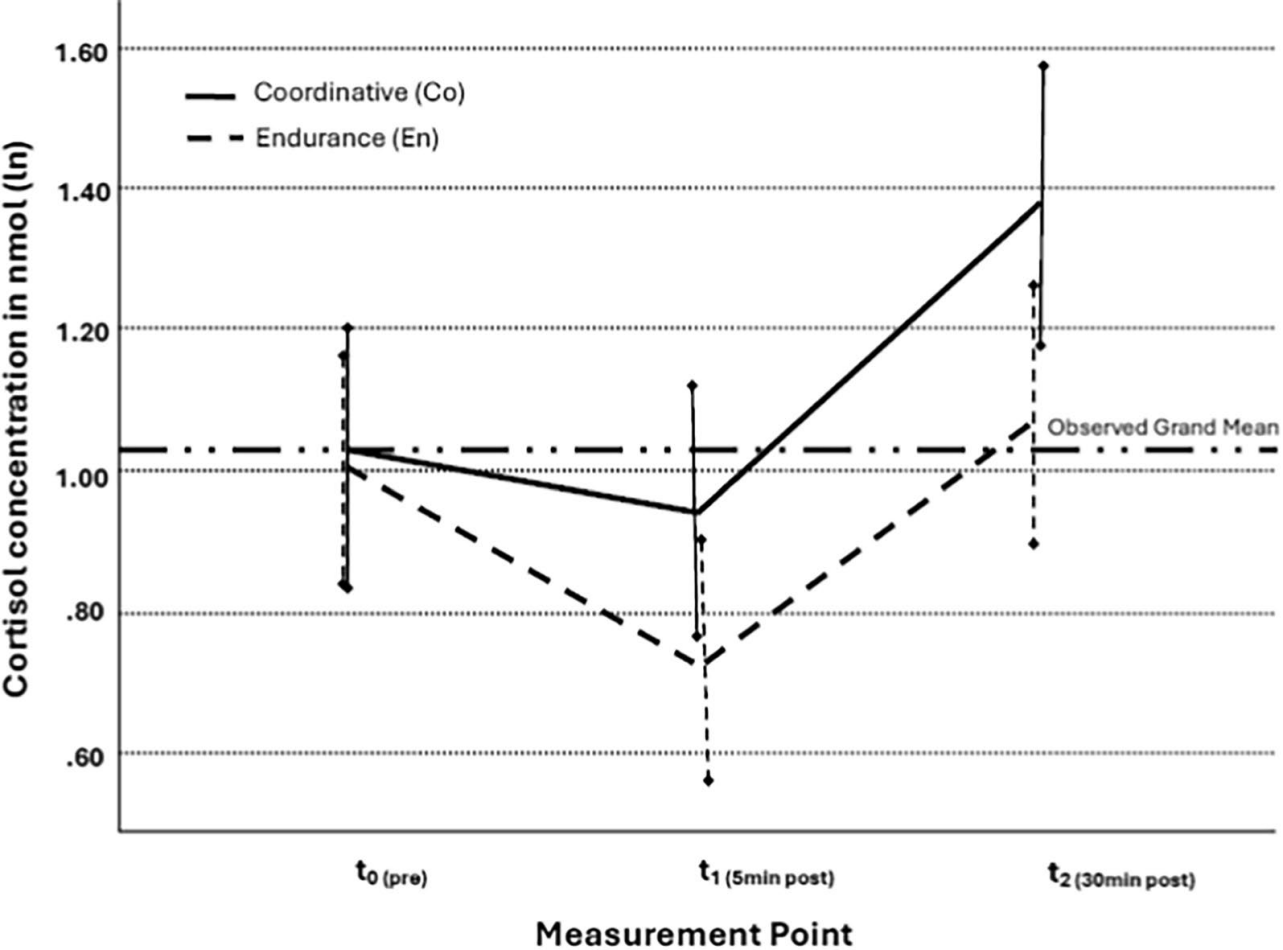
Acute changes in cortisol concentration in nmol/l (ln) before and after the coordinative (Co) compared to the endurance (En) interventions (t_0_: pre exercise, t_1_: 5 min post exercise, t_2_: 30 min post exercise)

Repeated measure ANOVA revealed a main effect of the measurement point (*F* (2) = 22.472, *p* < *0.001,* η^2^ = 0.290), and the type of exercise (*F* (1) = 5.587, *p* = 0.022*,* η^2^ = 0.092). The interaction of measurement points and type of exercise was also significant (*F* (2, 110) = 4.322, *p* = 0.016*,* η^2^ = 0.073, see Fig. 1).

Post hoc tests with Holm correction for multiple testing revealed that the cortisol values of t1 (*t* (55) = 2.097, *p* = 0.040, *d* = 0.741), and t2 (*t* (55) = 3.004, *p* = 0.004, *d* = 0.735), differed significantly between interventions with large effect sizes. Moreover, within Co, cortisol levels at t2 were significantly higher than in t0 (*t* (55) = − 3.858, *p* < 0.001, *d* = 0.684), and t1 (*t* (55) = − 6.814, *p* < 0.001, *d* = 0.484). In Co, cortisol levels at t0 and t1 did not differ significantly.

In En, cortisol levels significantly differed between t0 and t1 (*t* (55) = 4.000, *p* < 0.001, *d* = 0.519), and between t1 and t2 (*t* (55) = − 5.631, *p* < 0.001, *d* = 0.469) at medium effect sizes. Cortisol levels did not differ significantly between t0 and t2. The within conditions showed medium effect sizes.

## Discussion

It was assumed before conducting the study that stress measured by the cortisol concentration would be higher after the coordinative compared to the endurance exercise. We observed that more cortisol is released in the aftermath of acute coordinative exercise of moderate intensity (according to HR) than after an acute endurance exercise of the same intensity and duration. The endurance intervention was carried out at the same time seven days after the coordinative exercise, at between 64 and 76% HRmax lasting 15 min. These different exercise types can trigger distinct brain activity patterns. It has been suggested that automatic motor behaviors, as they were requested during the 15 min of exercise without an emphasis on motor coordination (En), are controlled by the basal ganglia [24]. The higher the motor demand, the more prefrontal cortex activity is required during the execution of motor tasks [20]. Coordinative exercise is known to also involve an activation of the cerebellum which besides motor functions [9] influences a variety of neurobehavioral systems including cognitive functions [14].

We explain our acute results with the effect we observed when comparing different types of chronic interventions (cortisol awakening response before and after a training intervention targeting endurance or coordination demands, three times a week over 10 weeks), which showed that coordinative training led to a greater reduction in cortisol concentration than endurance training [21]. According to Athanasiou et al. (2023) regular acute exercise leads to adaptations that are reflected in a chronic reduced physiological stress response [36]. It would therefore be expected that cortisol concentration is enhanced in an acute perspective but reduced after a chronic treatment. If this homeostatic threat is chronic it might lead to a lower response compared to the pretraining levels [37]. Rehfeld et al. (2018) observed a significant elevation in both white and gray matter, mainly in the cerebellum, after a coordinative compared to a non-coordinative training [38]. This also represents a lower training-induced concentration of the catabolic hormone cortisol, allowing tissue growth to proceed uninhibited. It is possible that different neurobiological signaling pathways are activated by different exercise interventions [21]. Coordinative intervention places more demanding cognitive requirements on movement accuracy than endurance exercise [24]. Budde et al. (2008) observed significantly higher attention after 10 min of coordinatively demanding exercise compared to endurance exercise [24]. Coordinative exercise requires perceptual and higher-level cognitive processes, such as attention, that are essential for mapping sensation to action and ensuring anticipatory and adaptive aspects of coordination [39]. This may show that coordinative interventions lead to a pre-activation of cognitive neuronal networks [24], which is reflected in endocrine responses. A significant increase in cortisol concentration observed 30 min after the end of the interventions was noted only in the Co condition compared to t0. This was also shown by a study not fully peer reviewed [40]. The researchers compared an acute single task with a dual task in university students and also reported a higher cortisol concentration in the saliva after the dual task (which included a higher coordinative effort). According to the literature, in adults, cortisol is first secreted a few minutes after the stressor before it then rises continuously until the peak of secretion is reached after approximately 20 to 30 min after the cessation of the exercise (which is not specified) [12]. The statistically non-significant results of the En condition could be shown in further data where cortisol was also not significantly elevated after 5 min resting post 15 min of acute endurance exercise in 14-year-olds (even though the exercise intensity was 65–75% HRmax) [17]. It therefore appears that the threshold after which an increased release of cortisol is reached is lowered by coordinatively demanding exercise. According to many studies, age is an important factor in this context [18]. However, we could not show significant differences between young and old in the average age we measured. In addition to the potential influencing factors already listed, particularly pronounced personality traits such as implicit affiliation motive or power motive [19, 41] could also be considered to affect the cortisol response. With regard to sex [18], there was no difference to be found in cortisol secretion in the present study. Due to our research question, we were unable to use a randomized controlled trial design. We first had to determine the HR during the coordinative exercise in order to control a comparable internal load for the second intervention. During the heterogeneous coordinative exercise intervention, it is more difficult to implement a constant stimulus (according to HR). The lack of random assignment is the major weakness of the quasi-experimental study design [42, 43]. However, this was unavoidable. The participants were exercising at an interindividual comparable intensity according to Garber et al. (2011) [26], and we calculated their HRmax using a formula [27] and did not explicitly test their HRmax for example, with the shuttle run test [44]. This was also due to the consideration that testing the HRmax in this population would probably have led to inaccuracies, since voluntary exhaustion is also associated with experience with movements with high exercise intensities and motivational factors. However, the questionnaire used generates a reliable measurement of motor fitness including the subscales cardiorespiratory fitness/endurance and gross motor coordination [31]. The PA-questionnaire (GLTQ) [29] we used is significantly associated with fitness of the participants [30].

## Conclusion

The novelty of this intraindividual study is that it shows for the first time that after an acute physical exercise intervention of moderate intensity (64–76% HRmax over a period of 15 min), cortisol levels increased more under the coordinative conditions than under the endurance conditions. These findings suggest that different types of acute exercise elicit distinct psychophysiological HPA axis responses, which could be valuable for future stress research and potentially applicable to clinical contexts involving stress responses, such as exercise in individuals with motor impairments.

## Data and material availability statement

The data that support the findings of this study are openly available in OSF at https://osf.io/nqbjx/?view_only=35d934417f9943108e9b42fa2c30c3d5.

## Abbreviations

ACTH: Adrenocorticotropic hormone
ANOVA: Analysis of variance
CRH: Corticotropin-releasing hormone
CO: Coordinative exercise
En: Endurance exercise
FFB: Questionnaire to Assess the Motor Function State—Fragebogen zur Erfassung des motorischen Funktionsstatus
GLTQ: Godin Leisure-Time Exercise Questionnaire
HPA: Hypothalamus-pituitary-adrenal
HRmax: Maximum heart rate
M: Means
PA: Physical activity
RPE: Rating of perceived exertion
SD: Standard deviations
UCLA: UCLA Loneliness Scale
